# Non-Immune Hydrops

**Published:** 1991-06

**Authors:** John Smoleniec, David James

**Affiliations:** RCOG Fetal Medicine Subspecialty Trainee, Department of Obstetrics, Bristol Maternity Hospital, Southwell Street, BS2 8EG; Consultant Senior Lecturer, University of Bristol

## Abstract

We report three cases of non-immune hydrops which were successfully treated in-utero. Each case had a different aetiology requiring a specific treatment.


### Non-Immune Hydrops. The Importance of Urgent Investigation if the Prognosis is to be Improved

**John Smoleniec, MRCOG**
RCOG Fetal Medicine Subspecialty Trainee, Department of Obstetrics, Bristol Maternity Hospital, Southwell Street, BS2 8EG.
**David James, MRCOG, MD,**
Consultant Senior Lecturer, University of Bristol.

### SUMMARY
We report three cases of non-immune hydrops which were successfully treated in-utero. Each case had a different aetiology requiring a specific treatment.

### INTRODUCTION
Hydrops fetalis (HF) is divided into two groups, isoimmune hydrops (IIH) and non-immune hydrops (NIH). The introduction of anti-D globulin in the early 1960's has led to a decrease in the number of IIH cases so that the NIH group accounts for up to 90% of all HF cases at present[1]. The prognosis for the NIH group has not decreased to the same extent as the IIH group because of the large number of possible causes as well as the large number of cases where no cause is found, which can be more than 50%[1]. Therefore there is no reliable maternal screening test to diagnose NIH early. The problem is compounded by the findings that anatomical and chromosomal abnormalities, which are not amenable to medical treatment, make up the majority of NIH cases, in caucasians[1,2]. However, with the improvement in sonography remarkable progress has been made in antenatal diagnosis, aetiology and therapy. These advances have shown the prognosis to vary greatly depending on the diagnosis. Therefore if the patient is to be properly counselled a thorough investigation of the mother and fetus is essential. We describe three cases of NIH detected antenatally, each with a different aetiology and each successfully treated in-utero.

### CASE 1
A 19 year old caucasian primigravida, at 30 weeks gestation, presented in premature labour. Clinically the fundal height was found to be large for dates and an ultrasound scan revealed polyhydramnios and fetal hydrops (skin oedema and bilateral pleural effusions). The patient was immediately transferred to the regional tertiary referral centre for further management. The mother and fetus were fully investigated according to established protocols and a working diagnosis of primary chylothorax was made. The pleural effusions were treated by creating pleuroamniotic shunts bilaterally using pigtail catheters sited under ultrasound control. The fetal hydrops resolved as did the polyhydramnios and premature labour. The patient was followed up on an outpatient basis. Labour was induced at 36 weeks gestation (5 weeks after pleuroamniotic shunting) when it was noticed that the right sided pleural effusion was reaccumulating. A thoracocentesis was performed before the patient was admitted to the labour ward. A 3160 gms live infant male was delivered vaginally with normal apgar scores. A further right sided thoracocentesis was performed by the paediatricians and headbox oxygen support was given for the first 24 hours. The infant's postnatal progress has been uneventful to date.

### CASE 2
A 29 year old caucasian woman at 31 weeks gestation in her second pregnancy was noted to have an intermittent fetal tachycardia at a routine antenatal visit whilst on holiday. No further investigations were advised. Ten days later whilst attending her local antenatal clinic the fetal tachycardia (greater than 240 beats per minute) was noted again. Ultrasound scan revealed fetal hydrops and the patient was sent to the regional tertiary referral centre for further investigations. Investigations revealed the fetal supraventricular tachycardia to be the only cause for the hydrops, the cardiac anatomy being normal.

The mother was started on antiarrhythmic therapy (flecainide), with the normal precautions being taken. The fetal arrhythmia reverted to and stabilised in sinus rhythm within 12 hours and the fetal hydrops resolved within 11 days. The antiarrhythmic agent was discontinued and the patient was discharged home with a portable fetal heart rate monitor for follow up at her local antenatal clinic. There was no recurrence of the tachyarrhythmia and a live infant girl was delivered vaginally at term with good apgar scores. Cardiological assessment was normal. At 3 months follow up, by the regional cardiologist, the infant was making normal progress and was discharged from further review.

### CASE 3
A 29 year old caucasian woman at 26 weeks gestation in her fifth pregnancy, having had four normal pregnancies previously, was found to be clinically large for dates at a routine antenatal clinic visit. Ultrasound scan revealed polyhydramnios and fetal hydrops (ascites only). There was no history of a recent flu-like illness or joint pains. The mother and fetus were fully investigated and fetal anaemia (Hb of 5.2 gm/dl) secondary to parvovirus B19 infection (parvovirus B19 particles were seen in the fetal blood using electron microscopy) was confirmed. An in-utero fetal blood transfusion was performed and the pregnancy was managed on an outpatient basis. Cordocentesis was performed on two separate occasions at 2 and 5 weeks post-transfusion, the fetal haemoglobin was found to be normal and there was no evidence of parvovirus. The fetal hydrops resolved within 3 weeks. The patient went into spontaneous labour at term and a live male infant was delivered vaginally with good apgar scores. Follow up of the infant to 1 year has been normal.

### DISCUSSION
These three cases demonstrate that with "early" in-utero diagnosis of fetal hydrops and prompt referral to a centre with the necessary expertise the outcome can be normal. The most important factors influencing prognosis are early antenatal detection (before the presence of oligohydramnios which is a poor prognostic indicator[3]), urgent in-utero investigation of the fetus and of the mother, to ascertain the aetiology of the NIH. These three cases were identified because of abnormalities on routine clinical examination. In two of the cases the fundal height was large for dates because of polyhydramnios, which has been reported to be present in up to 75% of cases of NIH[4], and an irregular fetal heart rate and rhythm was present in the other. Other clinical symptoms, signs and investigations associated with NIH include, a decrease in fetal movements, an abnormal cardiotocogram, maternal anaemia and metabolic diseases, preeclampsia and diabetes mellitus. It is only after full investigation of the NIH that the clinician will be in a position to

*Continued on p. 54*

### NON-IMMUNE HYDROPS,

*Smoleniec & James*

West of England Medical Journal Volume 106)ii) June 1991

*Continued from p. 43*

accurately counsel the parents and appropriately manage the pregnancy. Emergency caesarean section before full investigation is to be discouraged as the majority of cases of NIH are secondary to major anatomical and chromosomal abnormalities (which should be excluded first) and because in-utero treatment is the optimal management.

A 100% mortality was reported in one series when emergency caesarean section was performed for NIH without prior investigation nor in-utero treatment.[5] Although the outcome for these three cases was normal the expected prognosis is different for these three conditions. NIH caused by a supraventricular tachycardia has the best prognosis, a normal outcome in all six cases was reported by Carlton *et al*[2]. Whereas for congenital hydrothorax, which has been suggested as the primary cause of many of the unexplained cases of NIH[6], a 75% survival rate for an in-utero treated group has recently been reported[7].

NIH secondary to intra uterine infection is of a much lower incidence in comparison to the other two causes but a good prognosis can be expected if detected early.

### CONCLUSION

Recent reports[2,6,7] suggest that an ever improving prognosis can be expected, when NIH is diagnosed antenatally early in the disease process, and where major chromosomal and anatomical abnomalities have been excluded. If this trend is to be continued full investigation of all cases of NIH is mandatory.

### REFERENCES

1. WARSOF, S.L., NICOLAIDES, K.H. and RODECK, C.H. (1986) Immune and nonimmune hydrops. *Clin. Obstet. Gynecol.* **29**, 533.
2. CARLTON, D.P., MCGILLIVRAY, B.C. AND SCHREIBER, M.D. (1989) Nonimmune Hydrops Fetalis: A multidisciplinary Approach. *Clin. Perinatal.* **16**, 839–851.
3. NICOLAIDES, K.H., RODECK, C.H. and LANGE, I. *et al.* (1985) Fetoscopy in the assessment of unexplained fetal hydrops. *Br. J. Obstet. Gynaecol.* **92**, 671–679.
4. MACAFEE, C.A.J., FORTUNE, D.W. AND BEISCHER, N.A. (1970) Non-immunological hydrops fetalis. *J. Obstet. Gynaecol. Br. Commonw.* **77**, 226–237.
5. GOUGH, J., KEELING, J.W., CASTLE, B. and ILIFF, P.J. (1986) The obstetric management of non-immunological hydrops. *Br. J. Obstet. Gynaecol.* **93**, 226–234.
6. ROBERTS, A.B., CLARKSON, N.S., PATTISON, M.G. AND MOK, P.M. (1986) Fetal hydrothorax in the second trimester of pregnancy: successful intra-uterine treatment at 24 weeks gestation. *Fetal Ther.* **1**, 203–209.
7. RODECK, C.H., FISK, N.M., FRASER, D.I. and NICOLINI, U. (1988) Long-term in utero drainage of fetal hydrothorax. *N. Engl. J. Med.*, **319**, 1135–1138.

